# Neoadjuvant chemotherapy with paclitaxel plus cisplatin before radical surgery for locally advanced cervical cancer during pregnancy: A case series and literature review

**DOI:** 10.1097/MD.0000000000026845

**Published:** 2021-08-13

**Authors:** Huiqiong Huang, Yi Quan, Xiaorong Qi, Ping Liu

**Affiliations:** Department of Gynecology and Obstetrics, West China Second University Hospital, Sichuan University, Key Laboratory of Birth Defects and Related Diseases of Women and Children (Sichuan University), Ministry of Education, Chengdu, China.

**Keywords:** case report, locally advanced cervical cancer, neoadjuvant chemotherapy, pregnancy

## Abstract

**Rationale:**

Despite the development of human papillomavirus vaccines and significant improvement in cervical cancer screening over the past few years, cervical cancer remains the fourth most common cancer in women of childbearing age after breast cancer, melanoma, and thyroid cancer.

**Patient concerns:**

In this case report, the patients are all cervical cancer with stage IB2 and IB3 during pregnancy, the management constitutes a major medical challenge related to the impact of treatment on both maternal and fetal outcomes. Neoadjuvant chemotherapy (NACT) is an innovative option for cervical cancer patients with stage IB2 and IB3 before cesarean delivery and radical hysterectomy, and many chemotherapeutic agents are available, cisplatin plus paclitaxel yielded good maternal and fetal outcomes to the authors’ knowledge.

**Diagnoses:**

Masses were discovered in the cervix of 4 pregnant women with a history of vaginal bleeding. Biopsy examination of the masses revealed cervical carcinoma, which was staged in accordance with the International Federation of Gynecology and Obstetrics (i.e., FIGO) system.

**Interventions:**

The patients were treated with paclitaxel plus cisplatin, followed by cesarean delivery and radical hysterectomy.

**Outcomes:**

The 4 patients were treated successfully, with no recurrence during follow-up periods of 14 to 56 months, and all of the children were doing well with no anomalies.

**Lessons:**

Although further data are required, in pregnant women with invasive cervical cancer, NACT with cisplatin plus paclitaxel followed by cesarean delivery and radical hysterectomy was a practical treatment option.

## 1 Introduction

Cervical cancer is the leading cause of cancer-related death among women worldwide, with approximately 300,000 deaths annually (National Institute of Health. Cervical Cancer 2020). However, invasive cervical cancer during pregnancy is an extremely rare event, with an incidence between 0.05% and 0.1%.^[1]^ The birth rate for women >30 years of age has steadily increased over the past few decades, coupled with the fact that the peak age at most tumor occurrences is quite different than the peak age at pregnancy; as such, gestational tumors are relatively rare. When cervical cancer is diagnosed during pregnancy, successful treatment is possible if management is implemented by the collaboration of a multidisciplinary team of healthcare providers, which enables further optimization of maternal treatment while considering fetal development and providing psychological support and long-term follow-up to the infants.

The standard treatment for early stage cervical cancer is conization or simple hysterectomy, and that for locally invasive cervical cancer is concurrent chemoradiotherapy or neoadjuvant chemotherapy (NACT) followed by surgery.^[2–4]^ During pregnancy, radiotherapy and surgery can lead to spontaneous abortion, congenital malformations, and pediatric malignancies. Therefore, in pregnant women with cervical cancer, surgery must be delayed, and NACT may help prevent disease progression until the fetal lungs mature.^[5]^ In these cases, NACT is considered with subsequent cesarean delivery (CD) and radical hysterectomy (RH).^[6]^ NACT with cisplatin or a combination of cisplatin, vincristine, and bleomycin has been widely used. In non-pregnant cervical cancer patients, the response rates to NACT with cisplatin plus paclitaxel reportedly range from 40% to 50%.^[7]^ In one study involving 15 pregnant women with cervical cancer, NACT with cisplatin plus paclitaxel, followed by CD and RH^[8–14]^ yielded good maternal and fetal outcomes (Table 1).

**Table 1:** NACT with cisplatin plus paclitaxel in pregnant patients with cervical cancer.

Author	FIGO stage	NACT regimen	Type of surgery	Adjuvant therapy	Follow-up (mo)	Maternal outcome	Fetal outcome
Chun et al^[8]^	IB1	P + T	CD + RH + PLND + PALND	None	49	DOD	NL
	IB2	P + T	CD + RH + PLND + PALND	CT	60	NED	NL
Palaia et al^[15]^	IIB	P + T	CD + RH + PLND	None	10	NED	NL
Yousefi et al^[16]^	IB2	P + T	CD + RH + PLND + PALND	RT	6	NED	NL
Li et al^[17]^	IB2	P + T	CD + RH + PLND	CT + RT	21	NED	NL
	IB2	P + T	CD + RH + PLND	None	13	NED	NL
Fruscio et al^[18]^	IB2	P + T	CD + RH + PLND	None	113	NED	NL
	IB2	P + T	CD + RH + PLND	None	115	NED	NL
Kong et al^[19]^	IB1	P + T	CD + RH + PLND + PALND	None	104	NED	NL
	IB2	P + T	CD + RH + PLND + PALND	CT	35	NED	NL
	IB1	P + T	CD + RH + PLND + PALND	CT	24	NED	NL
Ricci et al^[20]^	IIA	P + T	CD + RH + PLND	CT + RT	63	DOD	NL
	IIA	P + T	CD + RH + PLND	RT	31	NED	NL
	IB2	P + T	CD + RH + PLND	None	27	NED	NL
	IB2	P + T	CD + RH + PLND	CT + RT	18	NED	NL

Herein, we report a case series of 4 pregnant women with invasive cervical cancer who were treated with NACT with cisplatin plus paclitaxel, followed by CD and RH. A review of the relevant literature is also presented and discussed.

## 2 Case presentations

The outcomes of 4 pregnant women with cervical cancer, who were treated with NACT in the authors’ hospital between January 2013 and January 2020, are reported (Table 2). These cases were staged according to the International Federation of Gynecology and Obstetrics (i.e., “FIGO”) system. The patients were carefully supervised and provided informed written consent to treatment. Fetal evaluation was managed through serial ultrasound assessments throughout the pregnancies (Table 2). The babies’ health status was assessed according to Apgar scores at 1, 5, and 10 minutes after birth. Follow-up data regarding the babies’ health and patient survival statuses were collected every 6 months. All 4 of the patients had stage IB disease and were treated with NACT with cisplatin plus paclitaxel, in accordance with current guidelines.^[9]^ All women underwent CD after completion of NACT. RH with bilateral salpingectomy and extensive lymph node resection was performed at the time of delivery.

**Table 2:** Clinical characteristics of the patients in our case series.

	Case 1	Case 2	Case 3	Case 4
Age (yr)	37	26	31	37
Obstetric history	G8P3 + 4	G4P1 + 2	G3P1 + 1	G2P0
Stage	IB1	IB1	IB2	IB2
Pathological diagnosis	SC	SC	SC	SC
GA at diagnosis (wk)	20	30 + 6	29 + 4	16 + 1
GA at start of NACT (wk)	21 + 3	31 + 4	32 + 5	20 + 1
NACT (mg/m^2^, courses)	P (50, 3), T (125, 3)	P (50, 1), T (125, 1)	P (50, 1), T (135, 1)	T (135, 3), C (AUC 4)
GA at delivery (wk)	35 + 3	34 + 5	36 + 4	36 + 3
Type of surgery	CD + RH + BS + PLND + PALND	CD + RH + BS + PLND + PALND	CD + RH + BS + PLND + PALND	CD + RH + BS + PLND + PALND
Response to NACT	LVSI + , N +	LVSI + , N-	LVSI + , N +	LVSI + , N-
Postoperative treatment	RT	RT	RT	RT
Neonatal weight (g)	2470	2330	2600	3300
Neonatal sex	Male	Female	Female	Female
Apgar scores	10–10-10	9–10-10	10–10-10	10–10-10
Follow-up (mo)	18	56	14	10
Maternal outcome	NED	NED	NED	NED
Fetal outcome	NL	NL	NL	NL

### 2.1 Case 1

**Figure 1:**
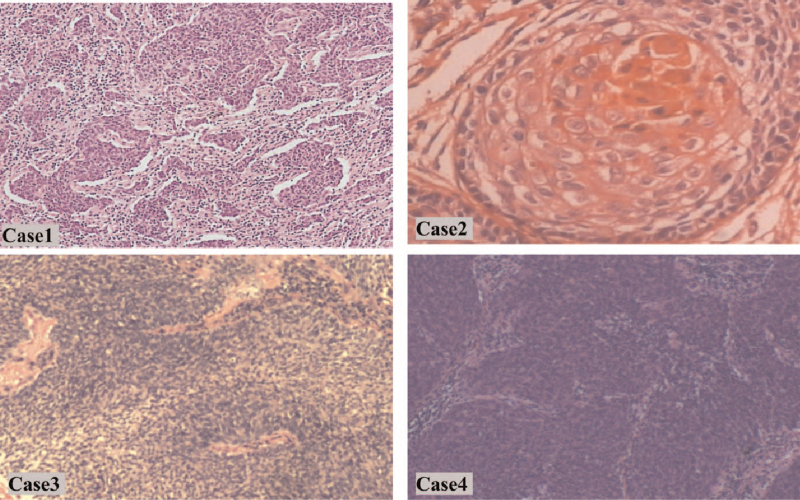
Histological examination of cervical tissue biopsy samples from Cases 1 (× 100), 3 (× 100), and 4 (× 100) revealed squamous cell carcinoma. Histological examination of cervical tissue biopsy samples from Case 2 (× 400) revealed mucinous adenocarcinoma cell carcinoma.

A 37-year-old woman was admitted to the authors’ hospital at 20 weeks’ gestation due to a one-month history of vaginal bleeding (Table 2). A mass measuring 2 cm in width was found in the cervix. Biopsy examination of the mass revealed cervical squamous cell carcinoma (Fig. 1). Invasive cervical cancer was diagnosed at stage IB1.

The patient underwent NACT with cisplatin (50 mg/m^2^) and paclitaxel (125 mg/m^2^) every 3 weeks starting at 21 + 3 weeks’ gestation, and 2 more cycles were initiated at 26 + 5 weeks and 30 weeks. She experienced no major adverse reaction, after chemotherapy, the local lesions of the cervix was partial response (RECIST 1.1) and subsequently underwent RH at the time of CD at 35 + 3 weeks. Neonatal birth weight was 2470 g, and Apgar scores at 1, 5, and 10 min were 10, 10, and 10, respectively. The baby exhibited no overt malformations.

Postoperative pathological examination of the resected tumor confirmed lymphovascular space involvement (LVSI) with positive lymph nodes (Fig. 3). Accordingly, radiotherapy was administered. No recurrence or metastasis was observed in this patient during 18 months of follow-up (Fig. 4) and her child was doing well.

**Figure 2:**
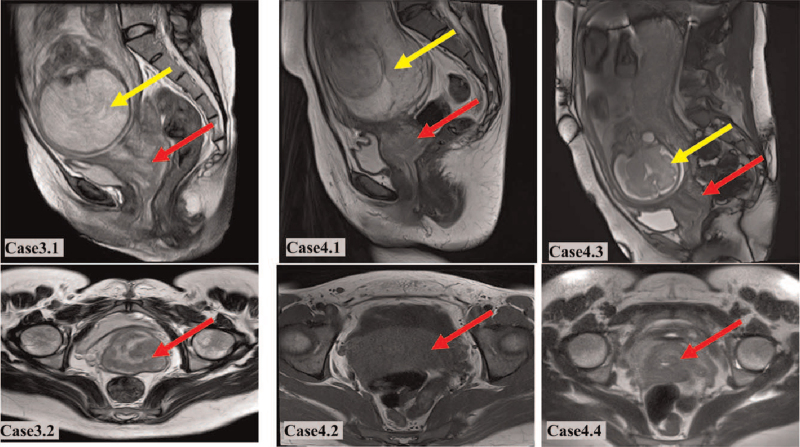
Case 3: T2 sagittal (3.1) and T2 coronal (3.2) pelvic magnetic resonance imaging (MRI) at 29 + 4 weeks of pregnancy reveals the fetus (yellow arrows) and tumor (red arrows). Case 4: T2 sagittal (4.1) and T2 coronal (4.2) pelvic MRI at 16 weeks and 29 weeks (4.3 and 4.4) of pregnancy reveals the fetus (yellow arrows) and tumor (red arrows).

**Figure 3:**
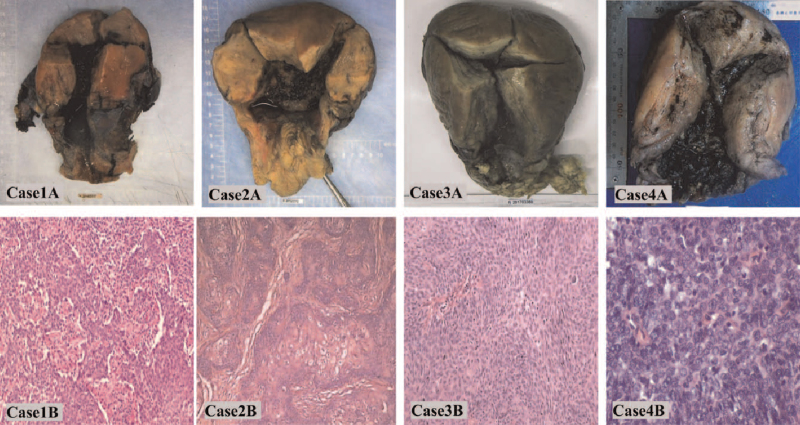
Case 1A-4A: Surgical specimen from the uterus and parametria after caesarean section and radical hysterectomy. Case 1B (× 100), 3B (× 100), 4B (× 400). Pathological images of hematoxylin and eosin stained samples revealing squamous cell carcinoma. Case 2B (× 100). Pathological images of hematoxylin and eosin stained samples revealing mucinous adenocarcinoma.

**Figure 4:**
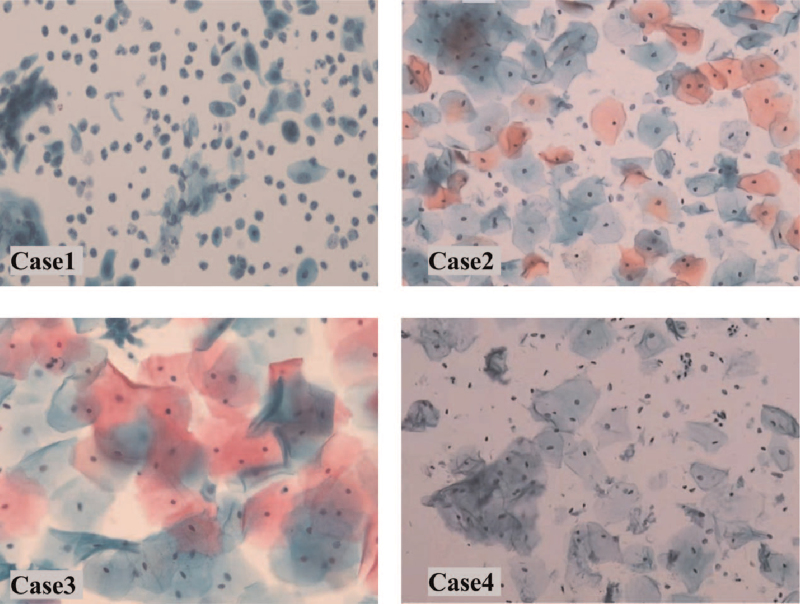
Cases 1-4. Images of liquid-based cytology testing of the vaginal cuff when followed-up recently.

### 2.2 Case 2

A 26-year-old woman was admitted to the authors’ hospital with vaginal bleeding at 30 + 6 weeks’ gestation (Table 2). The amount of vaginal blood loss was greater than that of normal menstrual blood loss. A cervical mass, measuring 3 cm in width, was detected on pelvic examination, and biopsy examination confirmed the presence of cervical mucinous adenocarcinoma (Fig. 1). T2 sagittal and T2 coronal pelvic magnetic resonance imaging (MRI) revealed the fetus and tumor at 29 + 4 weeks’ pregnancy (Fig. 2). Invasive cervical cancer was diagnosed at stage IB1.

The patient underwent treatment with NACT with cisplatin (50 mg/m^2^) and paclitaxel (125 mg/m^2^) at 31 + 4 weeks’ gestation. Because she was in the third trimester at the time cervical cancer was diagnosed, she completed only 1 cycle of NACT before surgery, and the local lesions of the cervix was SD (RECIST 1.1). The patient experienced no serious adverse reactions and then underwent RH at the time of CD at 34 + 5 weeks. Neonatal birth weight was 2330 g, and Apgar scores of the newborn at 1, 5, and 10 minutes were 9, 10, and 10, respectively. No anomalies were observed in the baby.

Postoperative pathological examination of the surgical specimen confirmed LVSI with no lymph node involvement (Fig. 3); therefore, radiotherapy was performed. No recurrence or metastasis occurred in this patient during the 56 months of follow-up (Fig. 4) and her child was healthy, with no anomalies.

### 2.3 Case 3

A 31-year-old woman presented with vaginal bleeding at 29 + 4 weeks’ gestation (Table 2). Pelvic examination revealed a mass, measuring > 4 cm in diameter, in the cervix. Biopsy examination of the mass confirmed cervical squamous cell carcinoma (Fig. 1). Invasive cervical cancer was diagnosed at stage IB2.

This patient was treated with NACT with cisplatin (50 mg/m^2^) and paclitaxel (135 mg/m^2^) at 32 + 5 weeks’ gestation. Because the cancer was diagnosed in the third trimester, the patient completed a single cycle of NACT before undergoing surgery, and the local lesions of the cervix was SD (RECIST 1.1). She did not experience adverse reactions and underwent RH at the time of CD at 36 + 4 weeks’ gestation. Neonatal birth weight was 2600 g, and 1, 5, and 10 min Apgar scores for the newborn were 10, 10, and 10, respectively. No anomalies were observed in the baby.

Postoperative pathological examination confirmed LVSI with positive lymph nodes (Fig. 3) and radiotherapy was performed. No recurrence or metastasis was detected in this patient during 14 months of follow-up (Fig. 4), and her baby exhibited normal growth and development.

### 2.4 Case 4

A 37-year-old woman, with no history of vaginal bleeding, underwent liquid-based cytology testing that revealed atypical squamous cells of undetermined significance (i.e., “ASCUS”) at 16 weeks’ gestation (Table 2). Pelvic examination revealed a mass in the cervix measuring > 2 cm in diameter. Biopsy examination of the mass confirmed cervical squamous cell carcinoma (Fig. 1). Invasive cervical cancer was diagnosed at stage IB2. This patient was treated with 3 cycles of NACT with cisplatin (50 mg/m^2^) and paclitaxel (135 mg/m^2^) every 3 weeks, which began at 20 weeks’ gestation and finished at 29 weeks. She experienced no adverse reactions and underwent RH at the time of CD at 36 + 3 weeks’ gestation. T2 sagittal and T2 coronal pelvic MRI revealed the fetus and tumor at 16 weeks and 29 weeks of pregnancy, respectively (Fig. 2), the cervical mass was partial response (RECIST 1.1) after 3 cycles of NACT. Neonatal birth weight was 3300 g, and 1, 5, and 10 minutes Apgar scores for the newborn were 10, 10, and 10, respectively. No anomalies were observed in the baby.

Postoperative pathological examination of the surgical specimen confirmed LVSI with no lymph node involvement (Fig. 3). Therefore, radiotherapy was administered. No recurrence or metastasis occurred in this patient during months of follow-up (Fig. 4) and her child was healthy, with no anomalies.

## 3 Discussion and conclusion

Ideally, treatment for cervical cancer during pregnancy should yield good outcomes for both mother and child. However, selecting a treatment strategy is challenging for both the patient and physician. The treatment of cervical cancer during pregnancy depends mainly on gestational age (first, second, and third trimester) at diagnosis, the extent of local invasion (stage and size of tumor), lymph node metastasis, tumor histological type, and the patient's wishes. The management of pregnancy complicated by cervical cancer follows the principle of individualization, which is based mainly on the 5 aspects of gestational age, tumor stage, grade, pathological type, and the patient's fertility desires at the time of diagnosis. Members of the International Network on Cancer, Infertility, and Pregnancy, in collaboration with other international experts, provided guidelines for gynecological cancers in pregnancy.^[21]^ According to these guidelines, simple cervical resection or wide conization is feasible for patients with stage IA2–IB1 disease, a tumor diameter <2 cm, and negative lymph nodes. Radical trachelectomy is not recommended during pregnancy.^[22]^ For pregnancy-preserving locally advanced cervical cancer, NACT or termination of pregnancy is recommended; NACT is the only option for patients who wish to continue the pregnancy until fetal maturity. Considering current clinical practices in China and the lack of adequate technology and experience, we recommend adopting a prudent attitude toward laparoscopic lymphadenectomy and cervical resection during pregnancy.

Chemotherapy should be administered before 35 weeks’ gestation to minimize the risk for transient neonatal myelosuppression, and to avoid maternal and fetal sepsis and hemorrhage. To reduce the risk for complications, delivery should be planned within 3 weeks after the last chemotherapy cycle.^[10]^ Drugs such as cisplatin, vincristine, and bleomycin have been used to treat cervical cancer during pregnancy, with cisplatin one of the most widely used. In 2 studies involving pregnant women with cervical cancer, cisplatin concentrations were significantly lower in the umbilical cord blood and amniotic fluid than in the maternal blood, and all newborns were healthy.^[14,23]^ This suggests that NACT with cisplatin can be safely used during pregnancy. Data regarding the safety of paclitaxel for the fetus are limited. A baboon model demonstrated that paclitaxel concentration in fetal blood was <2% of its maternal blood concentration.^[24]^ In addition, a retrospective study demonstrated the relative safety of paclitaxel during the second and third trimesters of pregnancy.^[17]^ However, the authors of that study emphasized that longer-term oncological and pediatric follow-up was necessary to confirm their findings.

In pregnant patients with early stage cervical cancer, 1 study reported that the risk for disease progression was relatively low. Most patients with stage IB disease experience satisfactory outcomes after planned treatment delay.^[25]^ Each case is unique, and the postponement of treatment should be carefully individualized. If fetal viability is achievable, pregnant women with cervical cancer who wish to continue their pregnancies and postpone treatment must be closely monitored and followed for a prolonged period. In such women, NACT may be a feasible method for managing cervical cancer during pregnancy.

To our knowledge, 21 cases of cervical cancer during pregnancy treated using NACT with platinum-based drugs have been reported in the literature; however, no specific/standardized regimen has been established.^[23]^ To date, when added to our 4 cases, 19 case reports have described pregnant women with cervical cancer undergoing NACT with cisplatin combined with paclitaxel before CD and RH (Table 1).^[8,15–20]^ Significant tumor regression was observed in these women. During delivery, RH was scheduled to occur between 33 and 36 weeks’ gestation when the fetal lung exhibited sufficient maturity. Among these 19 patients, 2 died. One patient with small cell neuroendocrine carcinoma who did not undergo postoperative adjuvant therapy died of widespread metastases 2 years after the diagnosis. Another patient with squamous cell carcinoma died of recurrence 1 year after the last cycle of platinum-based chemotherapy. In the remaining 16 patients who underwent NACT with cisplatin plus paclitaxel, the tumor did not recur.

We also reviewed the relevant literature over the past 20 years. We found that 31 pregnant women with cervical cancer were treated with NACT, cisplatin alone, cisplatin plus vincristine, bleomycin, or adriamycin. Follow-up data indicated that 3 patients were lost to follow-up, 7 died of tumor recurrence, and one was alive with disease.^[18,25–40]^

In conclusion, pregnant patients with cervical cancer treated with NACT with cisplatin plus paclitaxel, followed by CD and RH, experienced relatively good maternal and fetal outcomes. While primary surgery and radiotherapy can adversely affect fetal outcomes, the regimen we recommend for pregnant women with cervical cancer is an efficacious and feasible alternative. However, to date, few patients have received this treatment, and further studies with longer-term follow-up are required to confirm its safety and effectiveness during pregnancy.

## Author contributions

**Conceptualization:** Yi Quan, Ping Liu.

**Data curation:** Huiqiong Huang, Yi Quan.

**Formal analysis:** Huiqiong Huang, Yi Quan, Ping Liu.

**Investigation:** Huiqiong Huang, Ping Liu.

**Methodology:** Huiqiong Huang, Xiaorong Qi, Ping Liu.

**Project administration:** Huiqiong Huang, Xiaorong Qi.

**Resources:** Huiqiong Huang.

**Supervision:** Xiaorong Qi, Ping Liu.

**Writing – original draft:** Huiqiong Huang, Yi Quan.

**Writing – review & editing:** Xiaorong Qi, Ping Liu.
